# Supplementary material for: Practical application of quantum neural network to materials informatics
Source: Sci Rep. 2024 Apr 13;14:8583. doi: 10.1038/s41598-024-59276-0 (PMC11016107; doi:10.1038/s41598-024-59276-0)
Supplement: Supplementary file 1 — Supplementary Information. [file 41598_2024_59276_MOESM1_ESM.pdf]

Supplementary Information

Practical application of quantum neural network to materials informatics

Hirotoshi Hirai, Toyota Central R&D Labs., Inc., 41-1, Yokomichi, Nagakute, Aichi 480-1192, Japan
hirotoshih@mosk.tytlabs.co.jp

The melting point data for the metal oxides and the explanatory variables used in this study

mpid	formula_pretty	tmelt (°C)	formation_energy_per_atom (eV)	band_gap (eV)	density (g/cm^3)	cati_anio_ratio	dist_from_o (Å)	Ref.
mp-353	Ag2O	230	-0.322979072	0	7.163037726	2	2.058538362	[S1]
mp-1143	Al2O3	2025	-3.427314384	5.8537	3.873499119	0.666666667	1.98926705	[S1]
mp-306	B2O3	450	-2.798819686	6.3017	2.561902769	0.666666667	1.370723496	[S1]
mp-1342	BaO	1923	-2.824278854	2.0906	5.859229671	1	2.790391574	[S1]
mp-2542	BeO	2400	-3.103491949	7.4639	3.019437109	1	2.684602236	[S1]
mp-23262	Bi2O3	817	-1.662308663	2.1945	9.438175801	0.666666667	2.159104201	[S1]
mp-2605	CaO	2576	-3.306655539	3.6301	3.353083749	1	2.403555984	[S1]
mp-1132	CdO	1411	-1.365347709	0	8.100938389	1	2.360992779	[S2]
mp-20194	CeO2	1950	-3.929311298	1.8647	6.994759104	0.5	2.3674755	[S1]
mp-22408	CoO	1800	-1.285016756	0.588	5.451909946	1	1.963323187	[S1]
mp-19399	Cr2O3	1960	-2.367351145	0	5.167959274	0.666666667	2.024810985	[S1]
mp-7988	Cs2O	490	-1.189054108	0.5832	4.577833402	2	2.858819584	[S1]
mp-361	Cu2O	1222	-0.644929536	0.5127	6.204947168	2	1.838874747	[S1]
mp-2345	Dy2O3	2340	-4.010444631	3.9255	8.333958265	0.666666667	2.245100673	[S1]
mp-679	Er2O3	2418	-4.057857473	3.9629	8.894209986	0.666666667	2.213626286	[S3]
mp-1182469	Eu2O3	2050	-3.185170473	0	7.248568574	0.666666667	2.284912818	[S1]
mp-18905	FeO	1369	-1.071980789	0	6.162133081	1	2.132239765	[S1]
mp-19770	Fe2O3	1565	-1.707854729	0	5.143372283	0.666666667	2.783709404	[S4]
mp-19306	Fe3O4	1591	-1.645927057	0	5.108710523	0.75	1.92045638	[S1]
mp-886	Ga2O3	1725	-2.269760045	2.0078	5.887588	0.666666667	1.838445992	[S1]
mp-504886	Gd2O3	2330	-3.896175168	2.9411	7.632702113	0.666666667	2.291208135	[S1]
mp-733	GeO2	1115	-2.077921063	3.2501	4.264932176	0.5	1.752562202	[S1]
mp-352	HfO2	2774	-4.020659237	4.0165	10.24124329	0.5	2.130127501	[S1]
mp-812	Ho2O3	2415	-4.034431059	3.9458	8.612953628	0.666666667	2.228969657	[S3]
mp-22598	In2O3	1910	-1.997826769	0.9289	6.99871242	0.666666667	2.139235873	[S1]
mp-2723	IrO2	1100	-1.261741037	0	11.54830235	0.5	2.002264615	[S1]
mp-971	K2O	740	-1.248282652	1.7072	2.413811101	2	2.754967721	[S4]
mp-2292	La2O3	1840	-3.874906473	3.5319	5.904323249	0.666666667	2.411284057	[S1]
mp-1960	Li2O	1427	-2.062022012	4.854	1.969302476	2	2.015115164	[S2]
mp-1427	Lu2O3	2490	-4.12484172	4.0211	9.784622894	0.666666667	2.17244132	[S3]
mp-1265	MgO	2120	-3.054040299	4.4292	3.628903382	1	2.097001773	[S1]
mp-19006	MnO	1650	-1.979855143	0.1844	5.354813884	1	2.22404255	[S1]
mp-18759	Mn3O4	1560	-2.051136266	0.8571	4.886374082	0.75	1.924599183	[S1]
mp-18856	MoO3	759	-1.925526172	1.3714	4.482983053	0.333333333	1.717004827	[S1]
mp-2352	Na2O	1275	-1.438959774	1.8736	2.494753645	2	2.37506681	[S2]
mp-581967	Nb2O5	1460	-3.035114056	1.9254	4.300420662	0.4	1.843595226	[S1]
mp-1045	Nd2O3	2272	-3.782310718	3.708	6.487107728	0.666666667	2.361909662	[S1]
mp-19009	NiO	1552	-1.218721447	2.3009	6.762154478	1	2.093168941	[S1]
mp-551905	OsO4	40.1	-1.528748027	3.2472	4.389479957	0.25	1.722741229	[S1]
mp-2452	P2O5	569	-2.439768724	5.2021	2.71642236	0.4	2.403067553	[S1]
mp-20878	PbO	870	-1.450749967	2.1565	9.142133143	1	2.474224151	[S1]
mp-1336	PdO	750	-0.790462552	0	8.117605864	1	2.033291539	[S2]
mp-1018886	PdO2	200	-0.728374931	0	7.144507572	0.5	1.995027509	[S2]
mp-1285	PtO2	450	-0.927451319	0.6464	11.64897938	0.5	2.017884275	[S1]
mp-1394	Rb2O	400	-1.130422885	1.3192	4.015077324	2	2.921027596	[S2]
mp-1016092	Re2O7	296	-2.027655042	2.3646	5.837607745	0.285714286	2.669826276	[S1]
mp-725	RhO2	1050	-1.219802924	0	7.200741172	0.5	1.972645858	[S2]
mp-1716	Rh2O3	1100	-1.07218462	0.5597	8.286979112	0.666666667	2.063558723	[S2]
mp-554791	RuO4	25.4	-1.182991517	2.2052	3.612849511	0.25	1.688465207	[S2]
mp-2136	Sb2O3	655	-1.752500878	2.2239	5.832523086	0.666666667	1.996885848	[S1]
mp-216	Sc2O3	2485	-3.969496017	3.8235	3.818904932	0.666666667	2.087502873	[S2]
mp-726	SeO2	245	-1.15499177	3.2874	4.137816736	0.5	1.807503623	[S1]
mp-546794	SiO2	1696	-3.268032089	5.6896	2.223612883	0.5	1.612340901	[S1]
mp-218	Sm2O3	2300	-3.862715843	4.0477	7.079480081	0.666666667	2.322227054	[S1]
mp-856	SnO2	1385	-2.108330201	0.6519	6.873191556	0.5	2.069163919	[S1]
mp-2472	SrO	2430	-3.077726993	3.2748	4.987595381	1	2.583784408	[S1]
mp-1539317	Ta2O5	1872	-3.336480743	2.3196	7.564873671	0.4	2.735423005	[S1]
mp-1056	Tb2O3	2410	-3.984343459	3.9006	8.013812686	0.666666667	2.26043777	[S2]
mp-2125	TeO2	732.6	-1.495550845	2.2279	5.649784342	0.5	1.902089474	[S1]
mp-643	ThO2	3390	-4.368638369	4.4187	9.981284075	0.5	2.425286404	[S2]
mp-554278	TiO2	1720	-3.50248625	2.6774	3.615939061	0.5	2.667775524	[S1]
mp-458	Ti2O3	717	-3.304203011	0	4.602205289	0.666666667	2.067409135	[S1]
mp-1767	Tm2O3	2341	-4.093502702	3.8684	9.150946298	0.666666667	2.200393144	[S2]
mp-1597	UO2	2176	-3.751292436	0	11.05527685	0.5	2.361655624	[S1]
mp-25279	V2O5	656	-2.289930598	2.2826	3.437530506	0.4	2.377869359	[S1]
mp-19443	WO3	1473	-2.185901059	0.8578	6.883890239	0.333333333	2.695846823	[S1]
mp-2652	Y2O3	2410	-3.971683454	4.0973	5.026122833	0.666666667	2.24709132	[S1]
mp-2814	Yb2O3	2435	-2.700184903	0	8.519750717	0.666666667	2.280049683	[S3]
mp-2133	ZnO	1975	-1.791535259	0.7227	5.704217807	1	1.972789305	[S1]
mp-2858	ZrO2	2430	-3.814465951	3.5322	5.777331384	0.5	2.155738183	[S1]

25.4

[S1] Schneider, S J., COMPILATION OF THE MELTING POINTS OF THE METAL OXIDES. United States: N. p., 1963.
[S2] https://pubchem.ncbi.nlm.nih.gov/
[S3] J. P. Coutures and M. H. Rand, Pure & Appl. Chem., Vol. 61, No. 8, pp. 1461-1482,1989
[S4] Lide, DR (ed.). CRC Handbook of Chemistry and Physics. 81st Edition. CRC Press LLC, Boca Raton: FL 2000, p. 4-66
